# Complete Mitochondrial Genome Sequence Structure and Phylogenetic Analysis of Choy Sum (*Brassica rapa* var. *parachinensis*)

**DOI:** 10.3390/ijms27020872

**Published:** 2026-01-15

**Authors:** Tingting Liu, Li’ai Xu, Ziwei Hu, Xingpeng Xiong, Xia An, Jiashu Cao

**Affiliations:** 1 Zhejiang Institute of Landscape Plants and Flowers, Zhejiang Academy of Agricultural Sciences, Hangzhou 311251, China; liutt@zaas.ac.cn (T.L.); anxia@zaas.ac.cn (X.A.); 2 Laboratory of Cell & Molecular Biology, Institute of Vegetable Science, Zhejiang University, Hangzhou 310058, China; xiongxingpeng1989@163.com; 3 Key Laboratory of Quality and Safety Control for Subtropical Fruit and Vegetable, Ministry of Agriculture and Rural Affairs, Collaborative Innovation Center for Efficient and Green Production of Agriculture in Mountainous Areas of Zhejiang Province, College of Horticulture Science, Zhejiang A&F University, Hangzhou 311300, China; xuliai@zafu.edu.cn; 4 Ningbo Academy of Agricultural Sciences, Ningbo 315040, China; ziweihu0226@163.com

**Keywords:** choy sum, *Brassica rapa* var. *parachinensis*, mitochondrial genome, structural characteristics, phylogenetic relationship

## Abstract

Choy sum (*Brassica rapa* var. *parachinensis*) is an important vegetable crop in Brassicaceae. However, its mitochondrial genome has not been well studied. In this study, Illumina and Nanopore sequencing technologies were combined to assemble the complete mitochondrial genome of choy sum. The mitochondrial genome is a circular molecule of 219,775 bp, with a GC content of 45.23%. A total of 60 genes were annotated, including 33 protein-coding genes (PCGs), 23 transfer RNA (tRNA) genes, 3 ribosomal RNA (rRNA) genes, and one pseudogene. A total of 466 RNA editing sites were identified in the PCGs. Codon usage analysis revealed that leucine (leu) was the most frequently used amino acid. Twenty-nine codons showed a relative synonymous codon usage (RSCU) value greater than 1. Most of these preferred codons ended with A or U. A total of 308 repetitive sequences were detected, including 136 dispersed repeats, 17 tandem repeats, and 55 simple sequence repeats (SSRs). Evolutionary analysis indicated that most mitochondrial genes are under negative selection. The highest nucleotide diversity detected in the *cox2* gene suggests that this gene could serve as a valuable molecular marker for mitochondrial research in the species. Homology analysis found 22 homologous fragments between the mitochondrial and chloroplast genomes of choy sum. These fragments total 13,325 bp, representing 6.06% of the mitochondrial genome. Phylogenetic analysis showed that choy sum is most closely related to *B. rapa* var. *purpuraria*. This study offers a genomic resource for genetic improvement and breeding of choy sum. It also provides molecular insights into the evolution of *Brassica* species.

## 1. Introduction

Mitochondria are essential organelles in eukaryotic cells, serving as the primary sites of energy metabolism. They generate ATP through oxidative phosphorylation and participate in critical biological processes, including metabolism, signal transduction, and stress responses. These functions play an indispensable role in plant growth and environmental adaptation [1]. Unlike the conserved mitochondrial genomes of animals, plant mitochondrial genomes exhibit remarkable structural complexity and diversity. Their sizes vary substantially, ranging from 66 kb [2] to 18.99 Mb [3]. Structurally, they display high diversity, existing in multiple forms including circular, linear, and branched configurations [4,5,6]. Plant mitochondrial genomes contain abundant repetitive sequences that mediate structural rearrangements and lead to the formation of chimeric genes. These chimeric structures enable the mitochondrial genome to serve as a reservoir for cytoplasmic male sterility factors, thereby influencing plant growth and development [7,8]. As an extranuclear genetic system, mitochondrial genomes possess characteristics of completeness, polymorphism, and semi-autonomy. While maintaining their own unique expression systems, they encode relatively few genes and produce a limited repertoire of proteins. Consequently, they require coordinated regulation with nuclear genes to maintain normal biological functions [9]. Recent advances in high-throughput technologies and reduced sequencing costs have significantly accelerated research on plant mitochondrial genomes. Since the initial report of the *Arabidopsis thaliana* mitochondrial genome in 1997 [10], an increasing number of plant mitochondrial genomes have been deposited in National Center for Biotechnology Information (NCBI; https://www.ncbi.nlm.nih.gov/, accessed on 12 November 2025), including those of important crops such as rice [11], maize [12], wheat [13], and cotton [14]. These genomic resources provide valuable materials for investigating plant evolution, genetic diversity, and cytoplasmic inheritance mechanisms, while offering unique perspectives for understanding the genetic basis of important agronomic traits in crops.

The Brassicaceae represents one of the most successful evolutionary radiations among angiosperms. This family not only includes the classic model organism *A. thaliana* but also provides crucial experimental systems for plant molecular biology, developmental biology, and evolutionary studies [15]. Within Brassicaceae, the genus *Brassica* holds the greatest economic importance and exhibits well-defined genetic relationships. It comprises three elementary species: *B. rapa* (AA genome, 2n = 20), *B. nigra* (BB genome, 2n = 16), and *B. oleracea* (CC genome, 2n = 18). Additionally, three amphidiploid species have been derived through natural hybridization and genome doubling: *B. napus* (AACC, 2n = 38), *B. juncea* (AABB, 2n = 36), and *B. carinata* (BBCC, 2n = 34) [16]. These six species and their genomic relationships are classically described by the “U-triangle hypothesis” model [17], which illustrates how the three amphidiploid species originated from pairwise hybridization and subsequent genome doubling among the three elementary diploid species.

Mitochondrial genome research in Brassicaceae has yielded substantial and systematic findings, providing important insights into the evolutionary mechanisms and functional characteristics of species within this family. The mitochondrial genome of *A. thaliana* is 366,924 bp in length and contains 57 genes [10]. Studies have demonstrated that the PPR protein PGN, localized to mitochondria in *A. thaliana*, regulates mitochondrial ROS homeostasis and thereby influences plant stress resistance [18]. Comparative analysis between the mitochondrial genome of *B. napus* and that of *A. thaliana* reveals that plant mitochondrial genomes are relatively conserved in coding regions, with most variations occurring in non-coding regions [19]. Mitochondrial genome comparison and evolutionary analyses further indicate that the cytoplasm of *B. juncea* primarily originated from *B. rapa* [20], while the mitochondrial genome of *B. carinata* was derived from *B. nigra* [21]. Moreover, comparative genomic studies demonstrate that mitochondrial genome are highly conserved across different genera and species of Brassicaceae, whereas non-coding regions exhibit substantial interspecific divergence. This characteristic makes the mitochondrial genome an ideal molecular marker for species identification, infraspecific differentiation, and higher-level phylogenetic reconstruction within Brassicaceae, providing reliable molecular evidence for taxonomic revision and evolutionary history reconstruction of this complex plant group.

Choy sum (*B. rapa* var. *parachinensis*) is an important cultivated variety widely grown in southern China [22]. This crop is primarily valued for its tender main and lateral bolts, which possess a crisp texture and distinctive flavor. These edible parts are rich in vitamin A, vitamin C, dietary fiber, and antioxidants, giving the vegetable high nutritional and economic value [23]. Through long-term domestication and selection, choy sum has developed diverse cultivars that exhibit significant variation in stress tolerance, yield, and quality traits [24]. Current research mainly focuses on agronomic trait improvement, cultivation techniques, and physiological metabolism [25,26,27]. However, studies on its mitochondrial genome remain limited. To address this research gap, we sequenced the complete mitochondrial genome of choy sum using combined Illumina and Nanopore sequencing technologies. This study represents the first comprehensive analysis of its mitochondrial genome architecture, RNA editing sites, codon usage bias, and repetitive sequences. Furthermore, we employed comparative genomics to investigate mitochondrial genome collinearity and evolutionary relationships with related Brassicaceae species. Selection pressure and nucleotide diversity analyses were also conducted to identify potential molecular markers. Our findings provide the first mitochondrial genomic resource for choy sum, offering valuable insights for its genetic improvement and germplasm conservation. This study also contributes to understanding the evolutionary relationships within Brassicaceae species.

## 2. Results

### 2.1. Structural Features and Functional Annotation of the Mitochondrial Genome in Choy Sum

**Figure 1:**
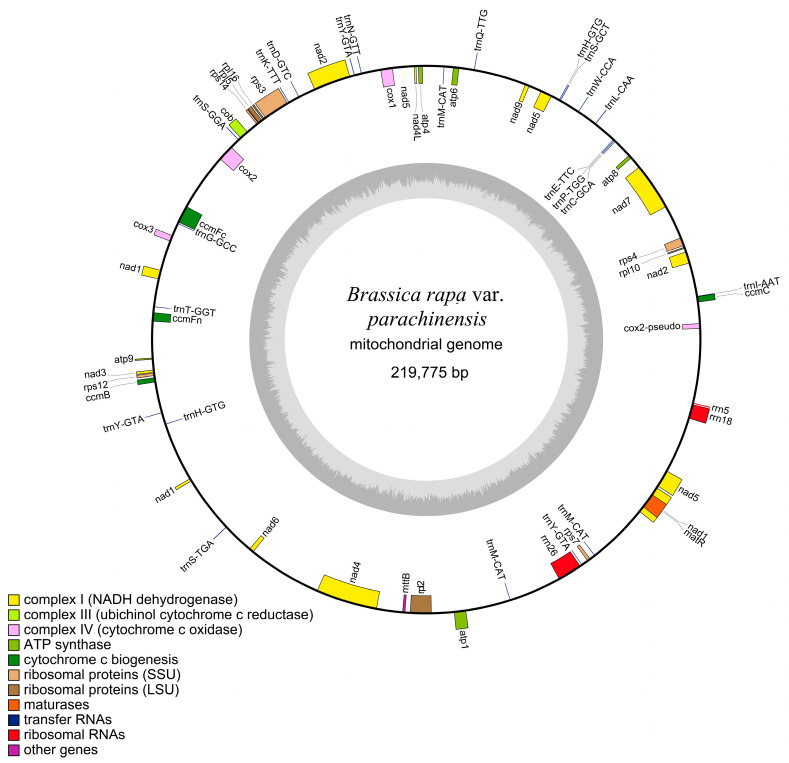
Map of the choy sum mitochondrial genome. In the circular map, genes encoded on the forward strand are located on the outer circumference, while those on the reverse strand are on the inner circumference. The gray inner ring represents the GC content. In the linear representation, forward-strand genes are depicted above the central line, and reverse-strand genes below it.

The mitochondrial genome of choy sum (PX776524.1) is a circular molecule of 219,775 bp with a GC content of 45.23% (Figure 1). Nucleotide composition analysis showed the following base distribution: adenine (A) = 27.45%, thymine (T) = 27.31%, guanine (G) = 22.32%, and cytosine (C) = 22.91% (Table 1). A total of 983 open reading frames (ORFs) were identified, and 60 genes were annotated, including 33 protein-coding genes (PCGs), 23 transfer RNA (tRNA) genes, 3 ribosomal RNA (rRNA) genes, and one pseudogene (Table S1). The total length of coding sequences was 29,055 bp, representing 9.55% of the genome. Among these, *nad5* was the longest gene (2010 bp), while *atp9* and *rpl10* were the shortest (225 bp each). The GC content of CDS regions was 42.63%. The tRNA sequences spanned 1728 bp (0.79% of the genome) with 51.62% GC content, while rRNA sequences totaled 5144 bp (17.70%) with 51.38% GC content (Table 1 and Table S1). These genes are primarily involved in essential mitochondrial functions, including ATP synthesis and cytochrome c biogenesis. Copy number analysis revealed single copies for 33 PCGs, 3 rRNA genes, and 15 tRNA genes. One tRNA gene was present in two copies, while two tRNA genes had three copies each. Additionally, 11 genes contained introns: four PCGs and two tRNA genes contained one intron each, one PCG contained three introns, and four PCGs contained four introns each (Table S1).

### 2.2. Codon Usage and Preference Analysis

Analysis of the mitochondrial genome identified 9652 codons encoding amino acids in choy sum. Leucine (Leu) was the most frequently encoded amino acid with 1053 codons, followed by serine (Ser, 856 codons) and isoleucine (Ile, 741 codons). Cysteine (Cys) showed the lowest usage with only 137 codons (Table S2). Most PCGs initiate with the standard ATG start codon. The exception is the *nad1* gene, which uses ACG as its start codon (with ATG as the edited version). Four types of stop codons were identified: TAG, TAA, TGA, and CGA (which edits to TGA) (Table S1).

**Figure 2:**
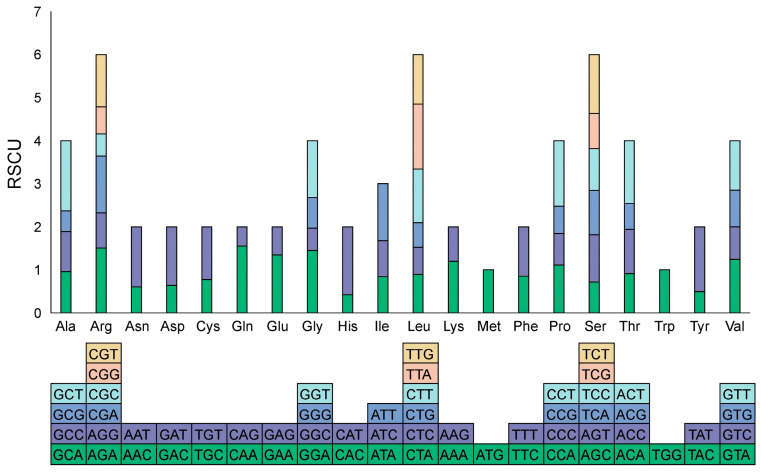
Relative synonymous codon usage (RSCU) analysis of the choy sum mitochondrial genome. The lower blocks correspond to all codons encoding each amino acid. The height of the upper bars denotes the cumulative RSCU value for all corresponding codons.

Analysis of relative synonymous codon usage (RSCU) revealed 29 codons with RSCU values greater than 1. Among these, 28 ended with A or U. Conversely, 33 codons showed RSCU values less than 1, with 28 of these ending in G or C. Only two codons exhibited RSCU values equal to 1. Tryptophan (Trp, UGG) and methionine (Met, AUG) are each encoded by a single codon, resulting in RSCU values of 1.0. The highest RSCU value was observed for GCU (Ala) at 1.6324, followed by CAU (His) at 1.5781 and CAA (Gln) at 1.5540. The lowest RSCU value was recorded for CAC (His) at 0.4219 (Figure 2; Table S2).

### 2.3. RNA Editing Site Analysis

RNA editing is an essential process for gene expression in plants. In this study, a total of 466 RNA editing sites were predicted across 33 PCGs (Table 2). Analysis of the relationship between gene length and the number of RNA editing sites revealed that longer coding sequences generally contain more editing sites, although no strict linear correlation was observed (Figure S1). All identified editing events resulted in amino acid changes. The most frequent substitution was CCG (Pro) to CTG (Leu), accounting for 78 of 466 sites. This was followed by TCA (Ser) to TTA (Leu), with 56 occurrences. The least frequent change was CGA (Arg) to TGA (stop codon), which was observed only once (Table 2).

Post-editing analysis revealed distinct changes in amino acid properties. At 53 sites, hydrophilic characteristics remained unchanged. A total of 203 sites transitioned from hydrophilic to hydrophobic properties, while 39 sites (8.37%) changed from hydrophobic to hydrophilic. Hydrophobicity was maintained at 170 sites (36.48%). These collective changes resulted in an overall increase in protein hydrophobicity. Additionally, one editing site (0.21%) introduced a premature stop codon from a hydrophilic amino acid (Table 2). The amino acid substitution patterns inferred from these predicted sites provide a preliminary reference for understanding potential functional modifications of mitochondrial proteins, but their actual occurrence and biological significance require experimental verification.

### 2.4. Analysis of Repetitive Sequences

**Figure 3:**
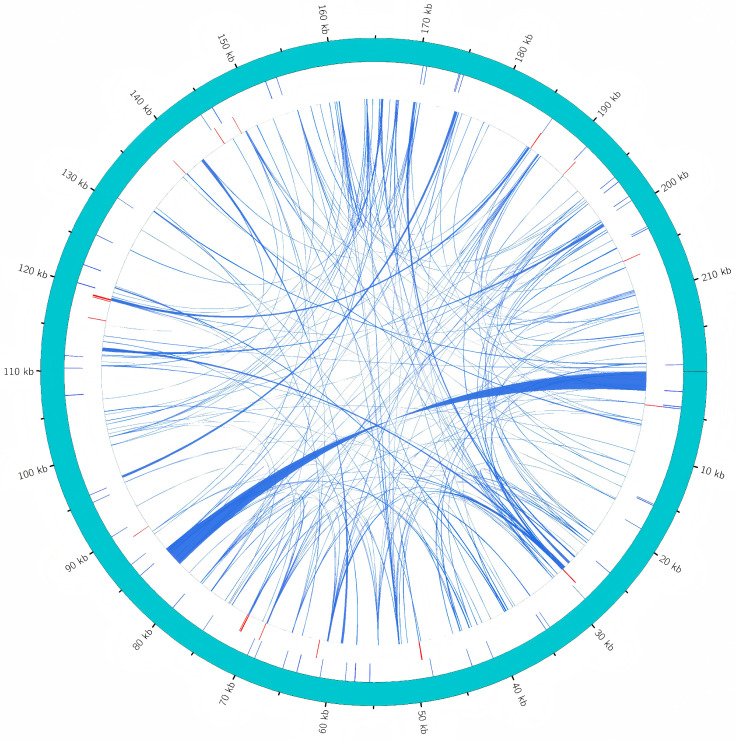
Distribution map of repetitive sequences in the mitochondrial genome of choy sum. The outermost track represents the mitogenome sequence, followed inward by the simple sequence repeats (SSRs), tandem repeats, and dispersed repeats.

Dispersed repeats are distributed throughout the genome in four main types: forward, palindromic, reverse, and complement repeats. In the mitochondrial genome of choy sum, 100 forward repeats and 136 palindromic repeats were identified. No reverse or complement repeats were detected. The total length of these repetitive sequences was 16,251 bp, accounting for 7.39% of the entire mitochondrial genome. The most abundant category consisted of repeats 30–39 bp in length, totaling 98 repeats (Figure 3; Table S3).

A total of 55 simple sequence repeats (SSRs) were identified in the mitochondrial genome of choy sum. Mononucleotide repeats were the most abundant type (20/55), followed by tetranucleotide (18/55), dinucleotide (11/55), trinucleotide (5/55), and pentanucleotide repeats (1/55). Among all 55 SSRs, the most abundant repeat motifs were A(10), T(10), TC(5), TTC(4), and AAGA(3), which corresponded to relative frequencies of 14.55% (8/55), 7.27% (4/55), 5.45% (3/55), 5.45% (3/55), and 5.45% (3/55), respectively (Figure 3; Table S4).

Tandem repeats, also known as satellite DNA, are characterized by repeating units of 1–200 bp with varying copy numbers. In this study, 17 tandem repeats were identified, all located in intergenic regions. These repeats ranged from 3 to 39 bp in length, with copy numbers varying from 1.9 to 8.3 (Figure 3; Table S5).

### 2.5. Selection Pressure and Nucleotide Diversity Analysis

**Figure 4:**
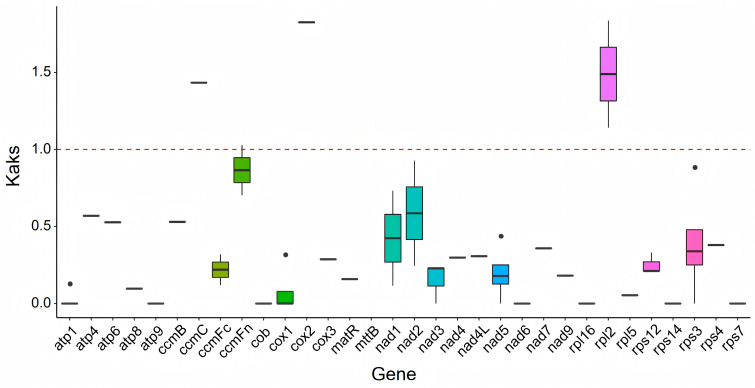
Ka/Ks analysis of mitochondrial genes across choy sum and seven Brassicaceae species.

Evolutionary selection pressure was assessed through the analysis of mitochondrial genomes from eight Brassicaceae species: choy sum, *A. thaliana*, *B. carinata*, *B. juncea*, *B. napus*, *B. nigra*, *B. oleracea*, and *B. rapa*. The average Ka/Ks ratio for 32 conserved PCGs was 0.34, indicating predominant purifying selection across most genes. This pattern suggests strong evolutionary constraints on mitochondrial PCGs in choy sum (Figure 4; Table S6). Notably, *ccmFn*, *ccmC*, *cox2*, and *rpl2* exhibited Ka/Ks ratios exceeding 1.0, suggesting they may have undergone positive selection during evolution.

**Figure 5:**
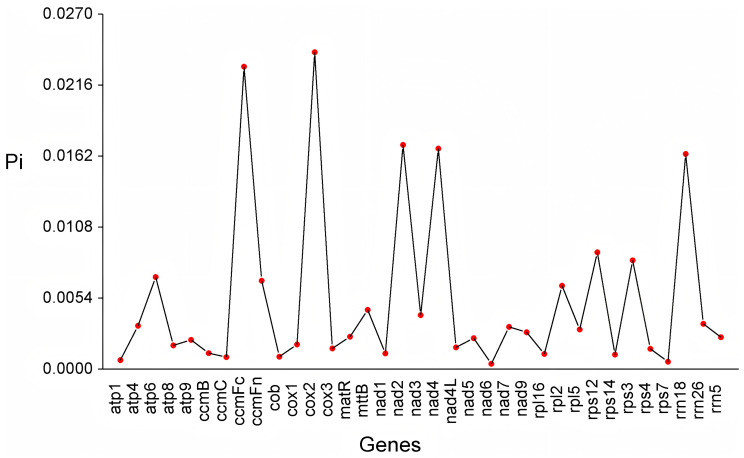
Nucleotide diversity (Pi) analysis of mitochondrial genome genes among eight Brassicaceae species.

Nucleotide diversity (Pi) was analyzed across the mitochondrial genomes of choy sum and seven Brassicaceae species: *A. thaliana*, *B. carinata*, *B. juncea*, *B. napus*, *B. nigra*, *B. oleracea*, and *B. rapa*. Pi values ranged from 0.0004 to 0.0241, with a mean value of 0.0053 (Figure 5; Table S6). Among the 35 genes exhibiting sequence variation, *rrn18* showed the highest number of variable sites (120), followed by *ccmFc* (81) and *cox2* (52). The genes *nad6*, *rpl16*, *rps14*, *rps7*, and *rrn5* contained the fewest variable sites, with only one each (Table S7). Five highly variable regions (Pi > 0.01) were identified: *cox2* (0.0241), *ccmFc* (0.0230), *nad2* (0.0171), *nad4* (0.0168), and *rrn18* (0.0164) (Figure 5). These regions represent potential molecular markers for mitochondrial genome analysis in choy sum.

### 2.6. Collinearity Analysis of the Mitochondrial Genome in Choy Sum

To investigate structural evolution of mitochondrial genomes within Brassicaceae, collinearity analysis between choy sum and related species was performed (Figure S2). The analysis revealed nearly complete coverage (100%) and predominantly continuous forward alignment with *B. juncea*, indicating strong conservation of both gene content and gene order between these two species. Similarly, 100% coverage was observed with *B. rapa*, though the collinearity pattern showed reverse-complementary alignment along the diagonal. This suggests the presence of sequence inversions between choy sum and *B. rapa* mitochondrial genomes. Comparative analysis with *B. oleracea*, *B. napus*, *B. carinata*, and *B. nigra* revealed high coverage (99.12%, 95.48%, 89.17%, and 89.14%, respectively) accompanied by multiple rearrangement events. Despite these structural rearrangements, core homologous blocks remained conserved across these species. In contrast, collinearity with *A. thaliana* was limited to 64.28% coverage, indicating substantial divergence in mitochondrial genome organization between these species.

### 2.7. Analysis of Homologous Sequences Between Mitochondrial and Chloroplast Genomes in Choy Sum

**Figure 6:**
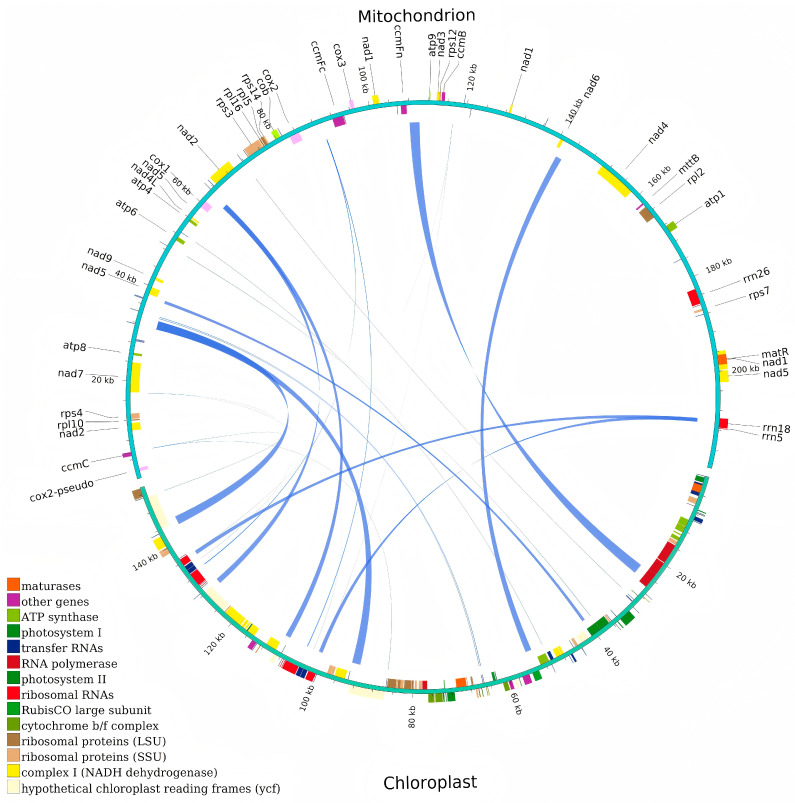
Homologous fragments identified between the mitogenome and chloroplast genome of choy sum. Genes from the same complex are denoted by blocks of identical color. The blocks in the outer and inner circles represent genes on the positive and negative strands, respectively. The junctions of the connecting lines indicate homologous sequences.

In choy sum, 22 homologous fragments spanning 13,325 bp were identified in both the mitochondrial and chloroplast genomes, accounting for 6.06% of the mitochondrial genome (Figure 6; Table S8). Length distribution analysis showed that 6 fragments exceeded 1000 bp, 7 fragments ranged between 100–1000 bp, and 9 fragments were shorter than 100 bp. Seven chloroplast genes were entirely located within these collinear regions: *ycf15*, *trnL*-*CAA*, *trnN*-*GUU*, *trnW*-*CCA*, *trnD*-*GUC*, *trnM*-*CAU*, *trnI*-*CAU*. Similarly, five mitochondrial genes were completely within the collinear regions: *trnL*-*CAA*, *trnN*-*GTT*, *trnW*-*CCA*, *trnD*-*GTC*, *trnM*-*CAT*. The predominance of tRNA genes among these shared sequences suggests that these genes may be more evolutionarily conserved than PCGs during organellar genome evolution.

### 2.8. Phylogenetic Analysis

**Figure 7:**
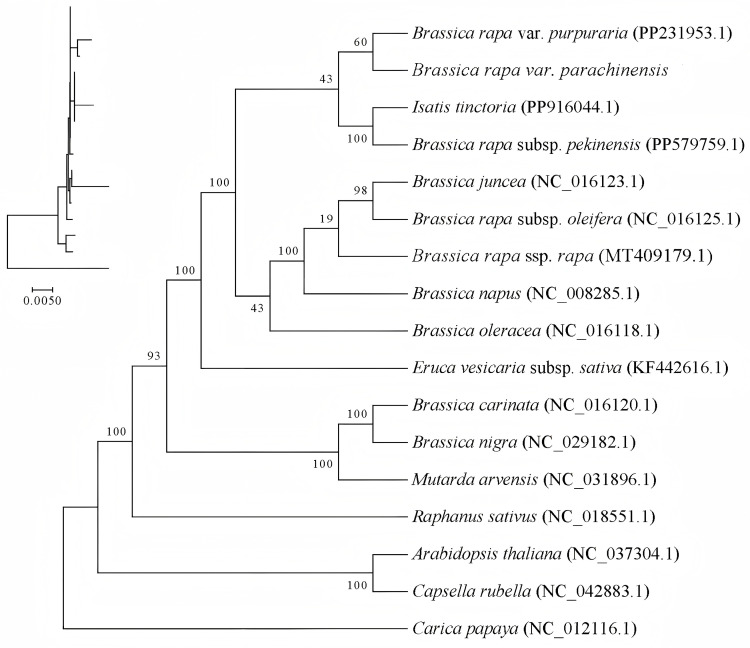
Phylogenetic analysis derived from mitochondrial genome sequences. The maximum-likelihood (ML) phylogenetic tree was constructed with 1000 bootstrap replicates, with the resulting support values indicated at each branch.

For a better understanding of the evolutionary relationships within Brassicaceae, mitochondrial genome data from 13 species was acquired from the NCBI database. A phylogenetic tree was constructed using the maximum likelihood (ML) method, with *Carica papaya* employed as the outgroup. The results show that choy sum clusters with *Brassica rapa* var. *purpuraria* and *B. rapa* in a single clade. Among these, choy sum exhibits the closest relationship with *B. rapa* var. *purpuraria*. The next closest relatives are other *Brassica* species, including *B. juncea* and *B. oleracea*, while *B. nigra* and *B. carinata* show more distant genetic relationships with choy sum. As expected, the outgroup species *C. papaya* demonstrates the most distant relationship (Figure 7).

## 3. Discussion

Mitochondria are double-membrane, semi-autonomous organelles found in most eukaryotic cells. They serve as essential sites for cellular respiration and energy conversion. Possessing their own genetic material and regulatory systems, these organelles play a vital role in cellular energy metabolism. These characteristics make mitochondria valuable tools for investigating eukaryotic evolution, genetic diversity, cultivar identification, and breeding programs [28]. In this study, the complete mitochondrial genome of choy sum was sequenced and analyzed. This analysis provided fundamental insights into its genomic organization, codon usage patterns, distribution of repetitive sequences, and phylogenetic relationships within the Brassicaceae family.

The mitochondrial genome of choy sum exhibits a typical circular structure with a total length of 219,775 bp. Comparative analysis revealed that this length represents approximately 60% (3/5) of the *A. thaliana* mitochondrial genome (366,924 bp) [10]. Interestingly, it shares identical length with another stem-use variety, *B. rapa* var. *purpuraria* [29], and differs by only 39 bp from the root-use subspecies *B. rapa* subsp. *rapa*) [30]. These observations suggest close size relationships among mitochondrial genomes within *B. rapa* species, with particularly high consistency between varieties of the same subspecies. The GC content of the choy sum mitochondrial genome is 45.23%. A total of 983 potential ORFs were identified, from which 60 functional genes were annotated, comprising 33 PCGs, 23 tRNA genes, 3 rRNA genes, and one pseudogene. The mitochondrial genome of the closely related *B. rapa* var. *purpurea* contains 977 ORFs and 59 functional genes. Its gene composition is nearly identical, differing only by one less pseudogene [29]. This further supports the high conservation of mitochondrial gene content among varieties within the same subspecies. In contrast, while *B. rapa* subsp. *rapa* exhibits similar GC content (45.24%), its mitochondrial genome contains significantly more genes—99 annotated genes including 78 PCGs, 18 tRNA genes, and 3 rRNA genes [30]. These comparative results demonstrate several key points. Choy sum, *B. rapa* var. *purpurea*, and *B. rapa* subsp. *rapa* all belong to the *B. rapa* species. However, the composition of their mitochondrial genes reveals a distinct phylogenetic pattern. Based on gene content, choy sum clusters more closely with *B. rapa* var. *purpurea*. In contrast, both show substantial divergence from *B. rapa* subsp. *rapa*. This pattern may reflect different domestication trajectories among these varieties. Additionally, one pseudogene was identified in the choy sum mitochondrial genome, which indicates potential functional degeneration during evolution. This phenomenon is not unique to choy sum, as similar observations have been reported in related Brassicaceae species such as *B. oleracea* var. *gongylodes* L. [31], potentially resulting from genomic reorganization or functional redundancy.

Codon usage bias refers to the non-random preference for specific synonymous codons during gene transcription and translation in particular organisms [32]. Analyzing codon usage patterns helps elucidate molecular mechanisms of biological adaptation and evolutionary relationships among species. Numerous studies have demonstrated that plant mitochondrial genomes preferentially use codons ending with A/U [33,34,35]. Our study confirms this pattern, with 28 of the 29 preferred codons ending in A or U, likely resulting from the combined effects of natural selection, mutation pressure, and genetic drift [36].

Beyond codon usage bias, RNA editing represents another crucial genetic regulation mechanism in plant mitochondrial genomes that influences gene product function. The number of RNA editing sites varies considerably among plant species. In this study, 466 RNA editing sites were identified across 33 PCGs in choy sum. This number is significantly lower than that reported in *Cinnamomum longepaniculatum* [37], but higher than in *Abelmoschus esculentus* [38]. Further analysis revealed that all RNA editing sites in choy sum involved C-to-U conversions. After editing, 43.56% of the affected amino acids changed from polar to hydrophobic, thereby enhancing protein hydrophobicity and potentially increasing protein stability. The identification of these RNA editing sites provides a foundation for investigating gene function evolution and predicting novel codons. However, due to the absence of experimental validation, the actual occurrence of predicted sites and their impacts on protein structure and function remain questionable. Therefore, further validation via RNA-seq or RT-PCR experiments is required in subsequent studies to clarify the actual occurrence of predicted sites, accurately quantify editing efficiency, and reveal the actual biological effects of these sites on the structural and functional modifications of mitochondrial proteins.

Repetitive sequences are widely distributed in plant mitochondrial genomes and play crucial roles in intermolecular recombination [39]. In this study, we identified 308 repetitive sequences, comprising 236 dispersed repeats, 55 SSRs, and 17 tandem repeats, collectively accounting for 7.39% of the total genome length. Among the dispersed repeats, only forward and palindromic types were detected, with no reverse or complementary repeats observed. This pattern has also been reported in other plant species such as *B. rapa* var. *purpuraria* [29], *Bromus inermis* [40] and *Indocalamus longiauritus* [41]. This suggests that dispersed repeat types in mitochondrial genomes may undergo convergent evolution across distinct plant families, though verification across more taxonomic groups is needed. Due to their high polymorphism and codominant inheritance, SSRs have been widely utilized for phylogenetic reconstruction, genetic diversity analysis, and species identification [42]. Among the 55 SSRs identified in the choy sum mitochondrial genome, mononucleotide repeats (predominantly A/T, 20 repeats) and tetranucleotide repeats (18 repeats) were the most abundant types. This result is consistent with the distribution characteristics of the dominant SSR types in the mitochondrial genome of *B. rapa* var. *purpuraria*, a con-specific variety of *B. rapa*, further reflecting the evolutionary conservation of repetitive sequences in the mitochondrial genomes among closely related varieties [29]. These SSR markers show potential for developing molecular tools for species identification and genetic map construction.

The Ka/Ks ratio serves as a valuable metric for assessing the impact of environmental stress on plant evolution [43]. The selection pressure analysis of 32 shared PCGs across eight Brassicaceae species revealed an average Ka/Ks ratio of 0.34, with 28 genes exhibiting Ka/Ks < 1. These results indicate that most PCGs in the choy sum mitochondrial genome are highly conserved. Notably, four genes (*ccmFn*, *ccmC*, *cox2*, and *rpl2*) showed Ka/Ks ratios > 1, which preliminarily suggests potential positive selection and adaptive evolution under environmental pressures. This pattern aligns with established evolutionary characteristics of plant mitochondrial genomes, where purifying selection acts as the dominant force to maintain gene function stability by eliminating deleterious mutations [44]. Nucleotide diversity (Pi) analysis provides insights into sequence variation across species [45]. Our results identified the *cox2* gene as having the highest Pi value among all examined regions, indicating its potential utility as a molecular marker for mitochondrial genome analysis in choy sum.

The transfer of chloroplast DNA fragments to mitochondrial genomes represents a widespread phenomenon in plants, leading to frequent occurrences of plastid-derived sequences in mitochondrial DNA [46]. A total of 22 chloroplast-derived DNA fragments in the mitochondrial genome of choy sum were identified, accounting for 6.06% of its total length. Comparative data reveal varying proportions of such sequences across species: 34 fragments (3.31%) in *Camellia hainanica* [33] and 35 fragments (1.78%) in *C. longepaniculatum* [37]. These findings demonstrate substantial interspecific variation in the extent of chloroplast-derived sequence integration among plant mitochondrial genomes. Notably, the transferred genetic material in choy sum predominantly consists of tRNA genes. This pattern suggests that these tRNA genes may exhibit higher evolutionary conservation compared to PCGs during interorganellar DNA transfer processes.

Collinearity analysis provides crucial insights into evolutionary relationships and genomic dynamics among related taxa [47]. The mitochondrial genomes of choy sum and *B. juncea* exhibit 100% coverage with continuous forward collinearity, providing preliminary evidence for high conservation between the AA genome of *B. rapa* (to which choy sum belongs) and the A subgenome of *B. juncea* at the mitochondrial level. Furthermore, although not complete, the mitochondrial genomes of choy sum show high collinearity with those of *B. oleracea*, *B. napus*, *B. carinata*, and *B. nigra*, all exceeding 89% coverage. This pattern indicates substantial collinearity retention among *Brassica* species, reflecting their close phylogenetic relationships. A similar phenomenon was reported by Xiao et al. [29] in their collinearity analysis between *B. rapa* var. *purpuraria* and *B. juncea*. In contrast, the mitochondrial genomes of choy sum and *A. thaliana* show only 64.28% coverage. This substantial structural divergence and weak collinearity suggest that these genomes have undergone extensive rearrangements and sequence variations during evolution, leading to significant collinearity decay.

A ML phylogenetic tree was constructed using mitochondrial genome sequences. The results show that choy sum, *B. rapa* var. *purpuraria*, and *B. rapa* subsp. *pekinensis*, form a distinct clade. Within this cluster, choy sum exhibits the closest relationship with *B. rapa* var. *purpuraria*, consistent with traditional taxonomic classification as both are varieties of *B. rapa*. Interestingly, the genetic relationship between *B. rapa* subsp. *pekinensis* and choy sum/*B. rapa* var. *purpuraria* appears closer than its relationship with *B. rapa* subsp. *oleifera* and *B. rapa* var. *rapa*. This phylogenetic pattern likely reflects different selection targets during human-directed domestication. However, this inference should be interpreted with moderation: the current phylogenetic analysis is solely based on mitochondrial genome data, and closely related Brassica species may be influenced by cytoplasmic introgression or convergent evolution. Future studies can integrate nuclear genome data or other organellar markers to enhance the robustness of phylogenetic inferences. Furthermore, *Brassica* species, including *B. napus*, *B. juncea*, and *B. oleracea* cluster with *B. rapa* species, while *B. nigra* and *B. carinata* form a separate cluster. This observation aligns with findings by Wang et al. [48] based on chloroplast genome analysis, who similarly reported that *B. oleracea* var. *alboglabra* clusters with *B. napus*, *B. rapa*, *B. juncea*, and *B. oleracea*, while *B. nigra* and *B. carinata* form a distinct group. These findings further support the classification proposed by Pradhan et al. [49], which divides *Brassica* crops into the *Brassica* lineage and the *Juncea* lineage.

## 4. Materials and Methods

### 4.1. Plant Materials, DNA Extraction, and Sequencing

The choy sum cultivar ‘Youqing 49’ was cultivated in the climate-controlled growth chamber of the College of Horticulture at Zhejiang A&F University (30°26′ N, 119°72′ E). In August 2024, fresh leaves from healthy plants were collected for DNA extraction using the HiPure Universal DNA Kit (D301, Genepioneer Biotechnologies, Nanjing, China). DNA quality was verified through 1.0% agarose gel electrophoresis. Qualified DNA samples were submitted to Genepioneer Biotechnologies Company for sequencing.

To obtain high-accuracy complete mitochondrial genome sequences, both short-read and long-read sequencing technologies were employed. Short-read sequencing assays were carried out on the Illumina NovaSeq 6000 platform (Illumina, San Diego, CA, USA, with paired-end reads set to a length of 150 bp. Raw sequencing datasets were refined through fastp v0.20.0 processing, thereby generating high-quality clean reads suitable for downstream analysis. Long-read sequencing assays were implemented on the Nanopore PromethION system (Oxford Nanopore Technologies, Oxford, UK), and subsequent data filtering was conducted with the aid of Filtlong v0.2.1.

### 4.2. Genome Assembly and Annotation

A multi-step assembly strategy was employed to reconstruct the complete mitochondrial genome. First, Long-read sequencing data underwent targeted alignment against a reference panel of plant mitochondrial core genes via the Minimap2 v2.1 software [50]. Sequences with alignments longer than 50 bp were retained as candidate sequences. From these, we selected the sequence exhibiting the highest number of aligned genes and optimal alignment quality as the initial seed sequence. Subsequently, the original long-read data were realigned to this seed sequence using Minimap2 v2.1 [50]. Sequences with overlaps exceeding 1 kb were incorporated into the seed sequence through an iterative alignment process, progressively expanding the mitochondrial genome dataset. The collected long-read data were then error-corrected using Canu [51]. Short-read sequence data underwent alignment against the corrected target sequence using Bowtie2 software version 2.3.5.1; this step was followed by hybrid assembly with Unicycler v0.4.8, which employed default parameters for the integration of both sequencing datasets. The assembly graph was visualized using Bandage v0.8.1. Finally, the corrected long-read data were mapped to the contigs generated in the second Unicycler step using Minimap2 v2.1 [50]. Branch orientations were manually determined to produce the final mitochondrial genome assembly of choy sum.

Annotations for PCGs and rRNA genes were determined by BLAST 2.16.0+ alignment against published plant mitochondrial reference sequences, with subsequent manual curation implemented using closely related species as a benchmark. The identification of tRNA genes was accomplished using tRNAscan-SE [52]. The mitochondrial genomic map was created by means of OGDRAW v 1.3.1 [53].

### 4.3. Analysis of RNA Editing Sites, Codon Usage Bias, and Repetitive Sequences

RNA editing sites were predicted using the PREP-Mt predictor. Codon usage bias was analyzed with a custom Perl script. SSRs were ascertained via MISA v1.0 [54] with the parameter configurations defined as follows: 1–10, 2–5, 3–4, 4–3, 5–3, and 6–3 repeat motifs. Tandem repeats underwent systematic detection utilizing Tandem Repeats Finder v4.09 [55]. Dispersed repeats were identified through BLASTN v2.10.1 alignments. All identified repeats were visualized using Circos v0.69-5 [56].

### 4.4. Analysis of Ka/Ks, Nucleotide Diversity, and Collinearity

Mitochondrial genomes of seven Brassicaceae species were downloaded from NCBI: *A. thaliana* (NC_037304.1), *B. rapa* (PP579759.1), *B. oleracea* (NC_016118.1), *B. napus* (NC_008285.1), *B. juncea* (NC_016123.1), *B. nigra* (NC_029182.1), and *B. carinata* (NC_016120.1). Alignment of homologous gene sequences was implemented by means of MAFFT v7.427 [57]. Ka/Ks analysis was performed with KaKs_Calculator v2.0 [58]. Nucleotide diversity (Pi) values per gene were determined via the DnaSP v5 program [59]. To achieve synteny analysis, we employed the nucmer software v4.0.0beta2 with the “–maxmatch” parameter to perform whole-genome alignment between the assembled mitochondrial sequence of choy sum and those of closely related species, and generated dot plots based on the alignment results.

### 4.5. Phylogenetic Analysis and Identification of Homologous Sequences Between Chloroplast and Mitochondrial Genomes

Mitochondrial genomes corresponding to 13 species within the Brassicaceae, along with *C. papaya*, were downloaded from NCBI. Multiple sequence alignment was carried out with the assistance of MAFFT v7.427 [57]. A ML phylogenetic tree was constructed with RAxML v8.2.10 [60] using 1000 bootstrap replicates. Homologous sequences spanning the chloroplast and mitochondrial genomes of choy sum were characterized through BLAST analysis, with the resulting data visualized via Circos v 0.69-5 [56].

## 5. Conclusions

This study presents the first comprehensive characterization of the mitochondrial genome in choy sum. The genome is a circular molecule of 219,775 bp with a GC content of 45.23%. We annotated 60 genes, including 33 PCGs, 23 tRNA genes, 3 rRNA genes, and one pseudogene. A total of 466 RNA editing sites were identified across the PCGs. Analysis of codon usage revealed 29 codons with RSCU values greater than 1, 96.55% of which ended with A or U. The genome contained 136 dispersed repeats, 17 tandem repeats, and 55 SSRs. Nucleotide diversity analysis identified *cox2*, *ccmFc*, *nad2*, *nad4*, and *rrn18* as highly variable regions. Evolutionary analysis indicated that the mitochondrial genomes of choy sum and related Brassicaceae species have predominantly undergone purifying selection, maintaining considerable conservation. Homology analysis detected 22 plastid-derived DNA fragments totaling 13,325 bp in the mitochondrial genome. Phylogenetic reconstruction confirmed the closest relationship between choy sum and *B. rapa* var. *purpuraria*. In summary, this research fills a critical gap in mitochondrial genomic resources for choy sum. The revealed genomic features, polymorphic regions, and evolutionary patterns provide new perspectives for understanding phylogenetic relationships within Brassicaceae species.

## Figures and Tables

**Table 1:** Nucleobase constitution of the choy sum mitochondrial genome.

Category of Sequence	Base Composition (%)						Size in bp (Proportion in Percentage)
	A%	T%	G%	C%	A + T%	G + C%	
Whole genome	27.45	27.31	22.32	22.91	54.77	45.23	219,775 (100%)
Protein-coding genes	26.34	31.03	21.7	20.92	57.37	42.63	29,055 (13.22%)
tRNA genes	22.57	25.81	28.76	22.86	48.38	51.62	1728 (0.79%)
rRNA genes	26.63	21.99	28.79	22.59	48.62	51.38	5144 (2.34%)

**Table 2:** Prediction of RNA editing sites in the choy sum mitochondrial genome.

Type	RNA-Editing	Number	Percentage
hydrophilic-hydrophilic	CAC (H) → TAC (Y)	6	
	CAT (H) → TAT (Y)	19	
	CGC (R) → TGC (C)	7	
	CGT (R) → TGT (C)	21	
	total	53	11.37%
hydrophilic-hydrophobic	ACA (T) → ATA (I)	6	
	ACC (T) → ATC (I)	2	
	ACG (T) → ATG (M)	6	
	ACT (T) → ATT (I)	8	
	CGG (R) → TGG (W)	21	
	TCA (S) → TTA (L)	56	
	TCC (S) → TTC (F)	21	
	TCG (S) → TTG (L)	41	
	TCT (S) → TTT (F)	42	
	total	203	43.56%
hydrophilic-stop	CGA (R) → TGA (X)	1	
	total	1	0.21%
hydrophobic-hydrophilic	CCA (P) → TCA (S)	7	
	CCC (P) → TCC (S)	6	
	CCG (P) → TCG (S)	5	
	CCT (P) → TCT (S)	21	
	total	39	8.37%
hydrophobic-hydrophobic	CCA (P) → CTA (L)	35	
	CCC (P) → CTC (L)	10	
	CCC (P) → TTC (F)	6	
	CCG (P) → CTG (L)	24	
	CCT (P) → CTT (L)	28	
	CCT (P) → TTT (F)	10	
	CTC (L) → TTC (F)	11	
	CTT (L) → TTT (F)	22	
	GCA (A) → GTA (V)	8	
	GCC (A) → GTC (V)	6	
	GCG (A) → GTG (V)	7	
	GCT (A) → GTT (V)	3	
	total	170	36.48%

## Data Availability

The data presented in this study are openly available from the NCBI at https://www.ncbi.nlm.nih.gov/nuccore/PX776524.1 (accessed on 4 January 2026), reference number PX240752.1.

## Supplementary Materials

The following supporting information can be downloaded at: https://www.mdpi.com/article/10.3390/ijms27020872/s1.

## Abbreviations

The following abbreviations are used in this manuscript:

ML	Maximum likelihood
NCBI	National Center for Biotechnology Information
PCGs	protein-coding genes
rRNA	ribosomal RNA
RSCU	Relative synonymous codon usage
SSRs	Simple sequence repeats
tRNA	transfer RNA
