# Supplementary material for: Complete Mitochondrial Genome Sequence Structure and Phylogenetic Analysis of Choy Sum (Brassica rapa var. parachinensis)
Source: Int J Mol Sci. 2026 Jan 15;27(2):872. doi: 10.3390/ijms27020872 (PMC12841004; doi:10.3390/ijms27020872)
Supplement: Supplementary file 1 [file ijms-27-00872-s001.zip › Supplementary Figure.pdf]

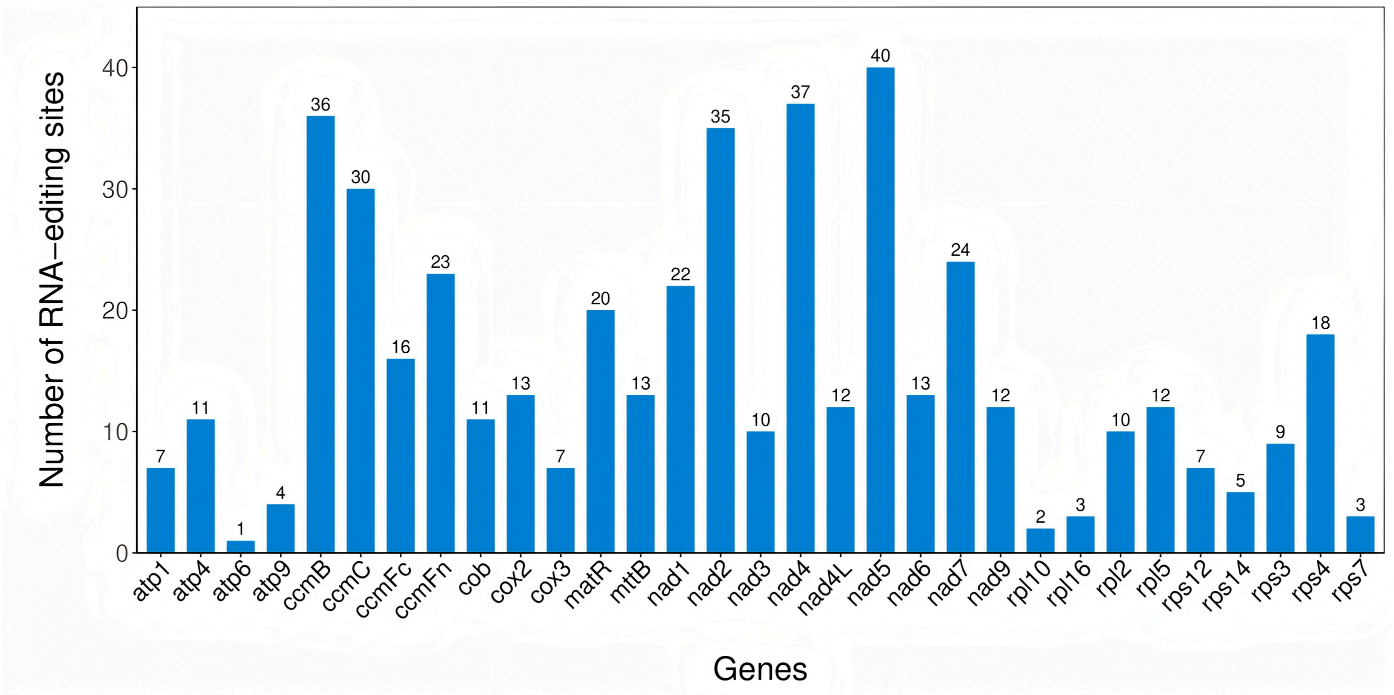

Figure S1. Statistic of RNA editing sites in 33 protein-coding genes (PCGs) present in the mitochondrial genome of choy sum.

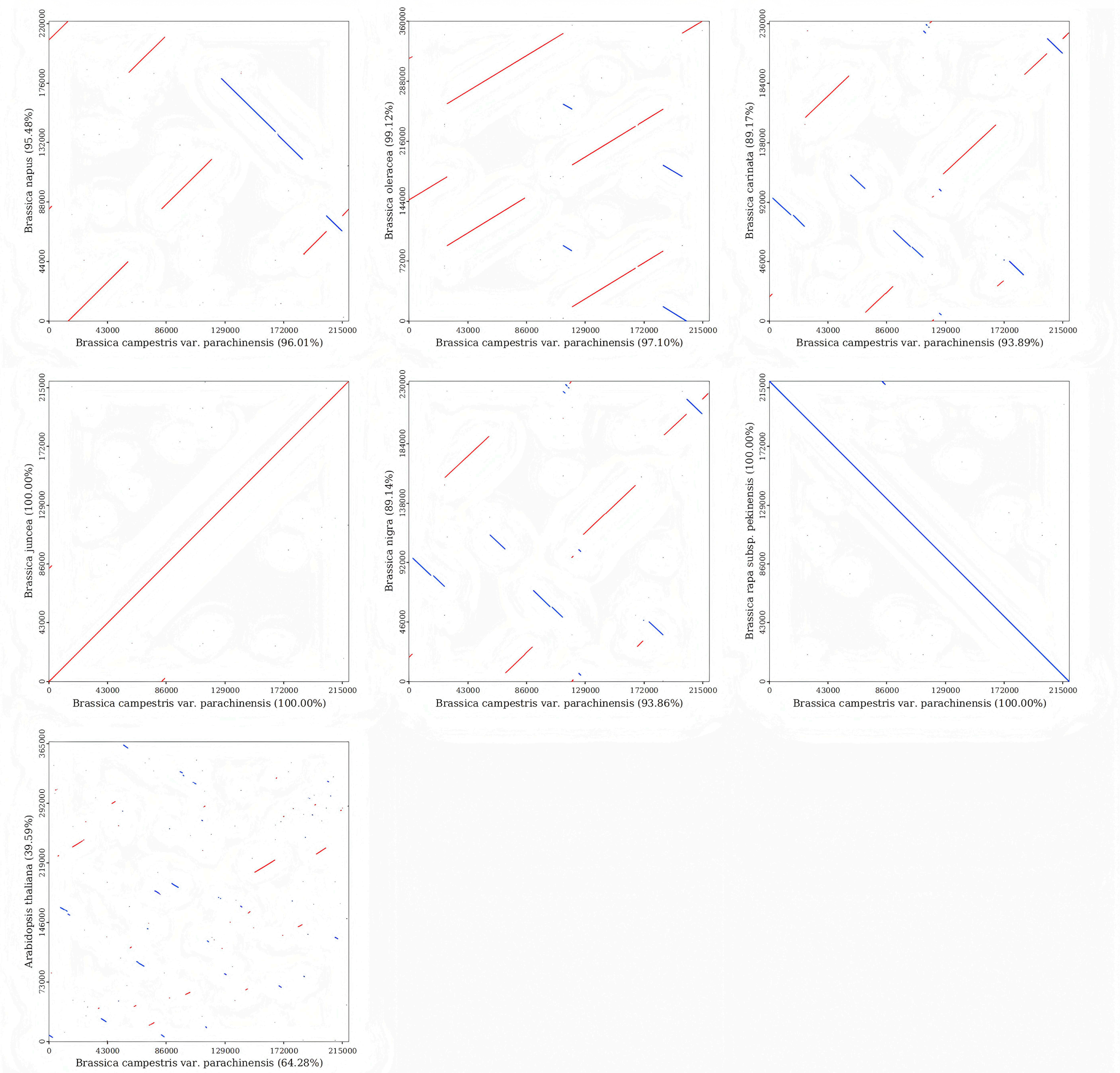

Figure S2. Collinearity analysis of mitochondrial genome sequences between choy sum and seven Brassicaceae species.
